# Calculation model and bearing capacity optimization method for the soil settlement between piles in geosynthetic-reinforced pile-supported embankments based on the membrane effect

**DOI:** 10.1371/journal.pone.0256190

**Published:** 2021-08-16

**Authors:** Zhen Liu, Aobo Zhang, Jiangping Xu, Cuiying Zhou, Lihai Zhang

**Affiliations:** 1 School of Civil Engineering, Sun Yat-sen University, Guangzhou, Guangdong, China; 2 Guangdong Engineering Research Center for Major Infrastructures Safety, Sun Yat-sen University, Guangzhou, Guangdong, China; 3 The University of Melbourne, Parkville, Melbourne, Australia; Al Mansour University College-Baghdad, IRAQ

## Abstract

The geosynthetic-reinforced pile-supported embankment (GRPSE) system has been widely used in road construction on soft soil. However, the application of the GRPSE system is often restricted by its high-cost. The reason is that they are designed for bearing control as defined in the past. During the construction process, the pile spacing is reduced to meet the requirements for the embankment bearing capacity and settlement. These factors cause the membrane effect to not be exploited. As a result, the utilization efficiency of the bearing capacity of the soil between the piles is low and the project cost is high. Therefore, in order to solve the problem of insufficient bearing capacity of soil between piles, we established a settlement calculation model of soil between piles based on membrane effect. The model considers the relationship between the geosynthetic reinforcement (GR) and the pile spacing. Based on the obtained model, a method for optimizing the soil bearing capacity of GRPSEs is proposed. By controlling the settlement of soil between piles, the bearing capacity of soil between piles and the membrane effect of embankment can be fully utilized. Therefore, the project cost can be reduced. Finally, the method is applied to field tests for comparison. The results show that the method is reasonable and applicable. This method can effectively exploit the membrane effect and improve the utilization efficiency of the bearing capacity of the soil between piles. An economical and reasonable arrangement scheme for the piles and GR was obtained. This scheme can not only ensure the safety of the project, but also fully utilize the bearing capacity of the soil between the piles and provide a new method for engineering design.

## 1. Introduction

A geosynthetic-reinforced pile-supported embankment (GRPSE) system consists of an embankment supported by geosynthetic reinforcement (GR) and piles with pile caps [1]. GRPSE represents a new of foundation treatment technology for construction of road infrastructure [2]. The arching effect and membrane effect due to different settlements of pile and soil are the fundamental working principles of GRPSE [3]. GRPSE system is widely used in highway and railway construction because it can greatly reduce the settlement of soft soil foundation under embankment load [4]. How to give full play to the bearing capacity of geosynthetic reinforced pile supported embankment (GRPSE) and produce better economic benefits is a important issue of current research.

Load sharing within components of a GRPSE system is a complex, and much research work has been carried out so far [5]. Chen et al. [4] investigated the soil arching effect in a GRPSE system and found that the soil arching effect of the embankment gradually increases with the increase of foundation settlement. By conducting field testing in conjunction with numerical simulation, Liu et al. [6] revealed that the load transfer between piles and the soil is significantly affected by the soil arching effect, and the amount of loading acting on the piles is approximately 14 times higher than that on the soil between piles. In addition, the study of Rui et al. [7] suggested that geosynthetics with a lower tensile stiffness produce a stronger arching effect than that with a higher tensile stiffness. To model the effect of GR, Van Eekelen et al. [8] proposed an analytical model based on BS8006—Code of practice for strengthened/reinforced soils and other fills, and they later on proposed a concentric arching model [3, 9] based on their previous research work. Zhuang and Ellis. [10, 11] analyzed the British standard BS 8006 published in 2010 and the 2012 amended version respectively through the finite element method. The results show that the BSI 2012 modified prediction is ‘best’ (but sometimes slightly unconservative), whereas the BSI 2010 modified prediction is conservative in all cases considered. Heitz et al. [12] proposed a theoretical method to investigate the mechanical behavior of various types of piles under dynamic loading considering the reduction coefficient of the soil arch and developed a model that reduces the soil arching, which in turn increases the strain in the GR. In addition, the study of Pham et al. [13] has demonstrated that the presence of GR could reduce the cumulative settlement, and cumulative settlement rate decreases with the increase of loading cycles. Furthermore, Xu et al. [14] found that increasing the cohesiveness of embankment fill soil could enhance the soil arching effect, and ultimately reduce the settlement of the foundation.

The bearing capacity of a GRPSE system depends on its settlement. While the studies of Ghosh et al. [15] and Fonseca et al. [16] showed that a higher tensile stiffness of GR could reduce the settlement of soft foundation. Hasan et al. [17] analyzed the reinforced body applied in different types of soil layers, the results show that the bearing capacity of geogrid applied in sand layer is higher than that in clay layer. The study of King et al. [18] shows that the additional settlements of end-bearing defective piles could result in a localized depression forming at the embankment surface. Experimental studies on the influence of the end bearing condition and modulus of piles on the performance of GRPSEs of Shen et al. [19] suggested that piles with relatively low modulus could increase the total settlement of bottom piles and foundation soil, and ultimately promote the lateral displacement of the embankment slope. In addition, many recent studies have extensively studies the critical factors that govern the load transfer mechanism and surface settlement of a GRPSE system, such as pile spacing, embankment height, pile cap size, GR stiffness and number of layers of geogrid [5, 20–23]. Furthermore, Wang and Mei [24] found that the micro-piles can improve the stability and deformation characteristics of the soft soil foundation. By studying the six commonly used GRPSE design methods, Wu et al. [25] found that current GRPSE design methods are relatively conservative. Zhanfang et al. [26] analyzed the settlement process of pile foundation, and thought that the dynamic problem can be transformed into static analysis, which provides reference for the design of bearing capacity of pile foundation. Liu et al. [2] proposed a comprehensive method to calculate the bearing capacity of pile soil with consideration of the pile-soil interaction. Zhuang & Wang. [27] found that cohesion is the most sensitive factor affecting the normalised maximum settlement and vertical stress of embankment.

In engineering design, GRPSEs are generally designed based on requirements for the bearing capacity. Firstly, the diameter of the pile is determined to make the bearing capacity of the foundation greater than the load. Then the layer-wise summation method is used to check whether the settlement can meet the requirements. The calculated settlement is only required to be less than a certain value. In the construction process, this is achieved by reducing the pile spacing to meet the requirements for the embankment bearing and settlement. As a result, the membrane effect cannot be exploited effectively, the utilization ratio of the bearing capacity of the soil between piles is low, and the project cost is high. Therefore, it is of great significance to establish an optimization method for the bearing capacity of soil between piles.

In this study, the Winkler elastic beam–foundation method is used to establish a calculation model for the soil settlement between piles based on the membrane effect. The optimization method of bearing capacity of soil between piles is proposed. The rationality of this method is analyzed through field tests. The results show that this method can effectively promote the membrane effect and improve the bearing capacity of soil between piles. This method can solve the problems of low utilization rate of foundation bearing capacity and high engineering cost. It has great reference value to improve the design method of GPRSEs.

## 2. Materials and methods

### 2.1. Calculation model for the soil settlement between piles

#### 2.1.1. The GRPSE system with single layer of geosynthetic reinforcement (GR)

**Fig 1:**
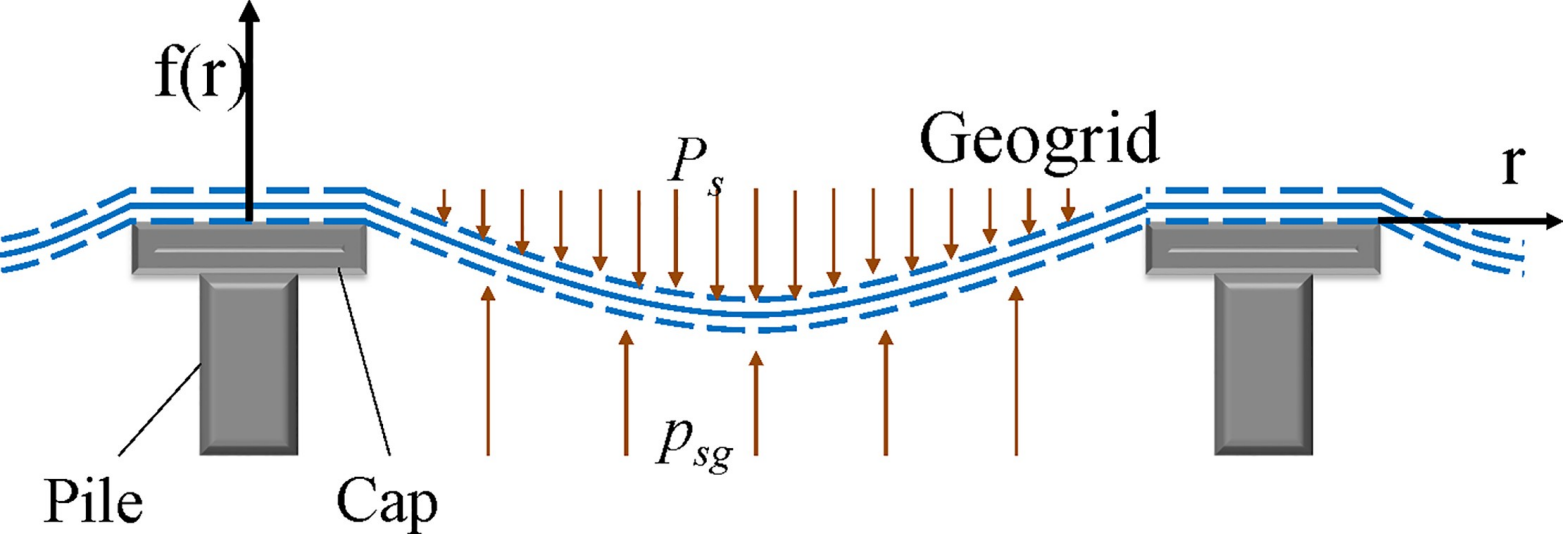
Schematic diagram showing stress of soil between piles for a GRPSE system with single layer of GR.

The total loading imposed on a GRPSE system is shared by the piles through the soil arching effect, soil between the piles and the GR. Fig 1 shows the schematic diagram of settlement of soil between piles for a GRPSE system with single layer of GR.

In addition, the following assumptions were made the GRPSE system investigated in this study.

- The deformation shape of soil between the piles is assumed to be parabolic;
- The deformation of GR is assumed be parabolic and stress in the GR can be modelled using Winkler theory [28];
- The mechanical properties of the soil within the GRPSE system is assumed to be isotropic;
- The mechanical behavior of the GR is assumed to be elastic;
- The settlement of the soil between piles is zero at the location of piles, and the stress of soil reaches to its maximum at the center of two piles.

Under small deformation of the GR, the shape of the geogrid can be simulated as a quadratic parabola. In a rectangular coordinate system with the pile center as the origin, the settlement deformation, *f*(*r*), of the GR can be defined as [29],

$$f(r)=ar^{2}+br+c(r_{p}<r<r_{e})$$ (1)

An axisymmetric approximation for the unit cell is used in this study. r_p_ is the radius of the pile cap and r_e_ is the equivalent treatment radius, r_e_ = S_a_/2 where S_a_ is the distance between piles.

Assuming the boundary conditions of *r* =*r*_*p*_,*f*(*r*) =0;*r* =*r*_*e*_,*f*(*r*) =*S*_*s*max_, the first derivative of the settlement *f*’(*r*_*e*_) = 0. Assuming *S*_*s*max_ is the maximum settlement of the soil between piles, we obtain,

$$\begin{array}{l} a=\frac{-S_{s\max}}{{\left(r_{e}-r_{p}\right)}^{2}}\\ b=-2ar_{e}\\ c=-ar_{p}^{2}-br_{p} \end{array}$$ (2)

By ignoring the horizontal displacement of the GR during the settlement process, the tensile strain of the GR at the edge of the pile cap can be determined as $\varepsilon_{g}=\sqrt{1+\tan^{2}\theta }-1$, where $\tan\theta =\frac{r_{e}}{S_{s\max}}$, and *θ* is the angle between the tangent line at the pile edge and the horizontal direction after the deformation of the GR. Let *E*_*g*_ is the elastic tensile modulus of the GR, the tensile force at the edge of pile cap *T* = *ε*_*g*_*E*_*g*_.

Assuming that the distance between piles (*S*_*a*_) is much larger than the settlement of the soil between piles (*i*.*e*. *S*_*a*_≫*S*_*s*max_), The soil stress between piles caused by the subsidence of the upper part of the GR (*p*_*sg*_, Fig 1) can be defined by using the Winkler elastic foundation–beam model. Winkler assumes that the settlement *s* (x, y) at any point on the interface of the foundation soil is proportional to the pressure (*p*_sg_). The settlement at this point has nothing to do with the pressure at other points. The function expression is: *p*_sg_(x,y) = k*s(x,y), where *k* is the coefficient of the subgrade soil [30]. According to the above assumptions, *f(r)* = *s*(x,y) can be obtained. Therefore, Eq (3) is expressed as follows:

$$p_{sg}(r)=-f(r)k.$$ (3)

where *k* is the coefficient of the subgrade soil between the piles of a GRPSE system. Assuming uniform distribution of upper covering soil and *P*_*s*_ is the stress of the soil, equilibrium equation describing the stress in soil between the piles in vertical direction can be determined as,

$$\pi (r_{e}^{2}-r_{p}^{2})p_{sg}+2\pi r_{p}T\sin\theta =P_{s}\pi (r_{e}^{2}-r_{p}^{2}).$$ (4)

Using Eqs (1), (2), (3) and (4), *S*_*s*max_ can be defined as,

$$S_{s\max}=\frac{P_{s}(r_{e}^{2}-r_{p}^{2})+\sqrt{{\left[P_{s}(r_{e}^{2}-r_{p}^{2})\right]}^{2}-16[E_{g}r_{p}^{2}(kr_{p}-kr_{e}r_{p}-E_{g})]}}{4(kr_{p}-kr_{e}r_{p}-E_{g})}.$$ (5)

#### 2.1.2. The GRPSE system with multi-layers of geosynthetic reinforcement (GR)

The stress and deformation of multi-layer GR(geogrid) and geocell is different from that of single-layer GR. In this study, a single layer GR in GRPSE system is modelled as a tensioned membrane due to its relatively low bending stiffness. Multi-layer GR system has the characteristics of bending due to the interlock of GR with the surrounding soil and the reinforced cushion. This makes the system composed of geogrid and sand cushion has large bending stiffness. When calculating the soil settlement between piles, the bending stiffness has a greater influence on the soil settlement between piles, while the tensile stiffness has a smaller influence on the soil settlement between piles [31, 32]. Under the action of gravity and load, the maximum deflection of GR is regarded as the maximum settlement of soil between piles, so this paper mainly calculates the maximum vertical deformation caused by the bending of GR, and uses the bending stiffness to calculate. Geocell has bending rigidity, which can be calculated by the same method as multi-layer geogrid [33].

**Fig 2:**
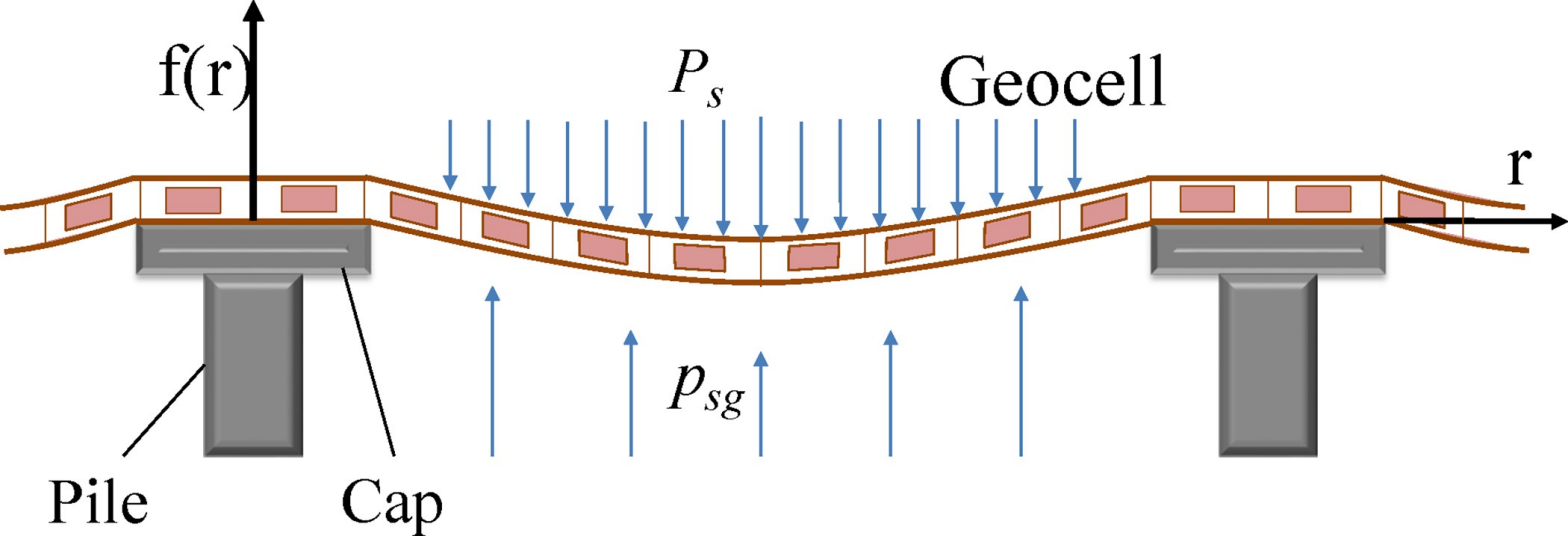
Schematic diagram showing stress of soil between piles for a GRPSE system with multi-layers of GR.

**Fig 3:**
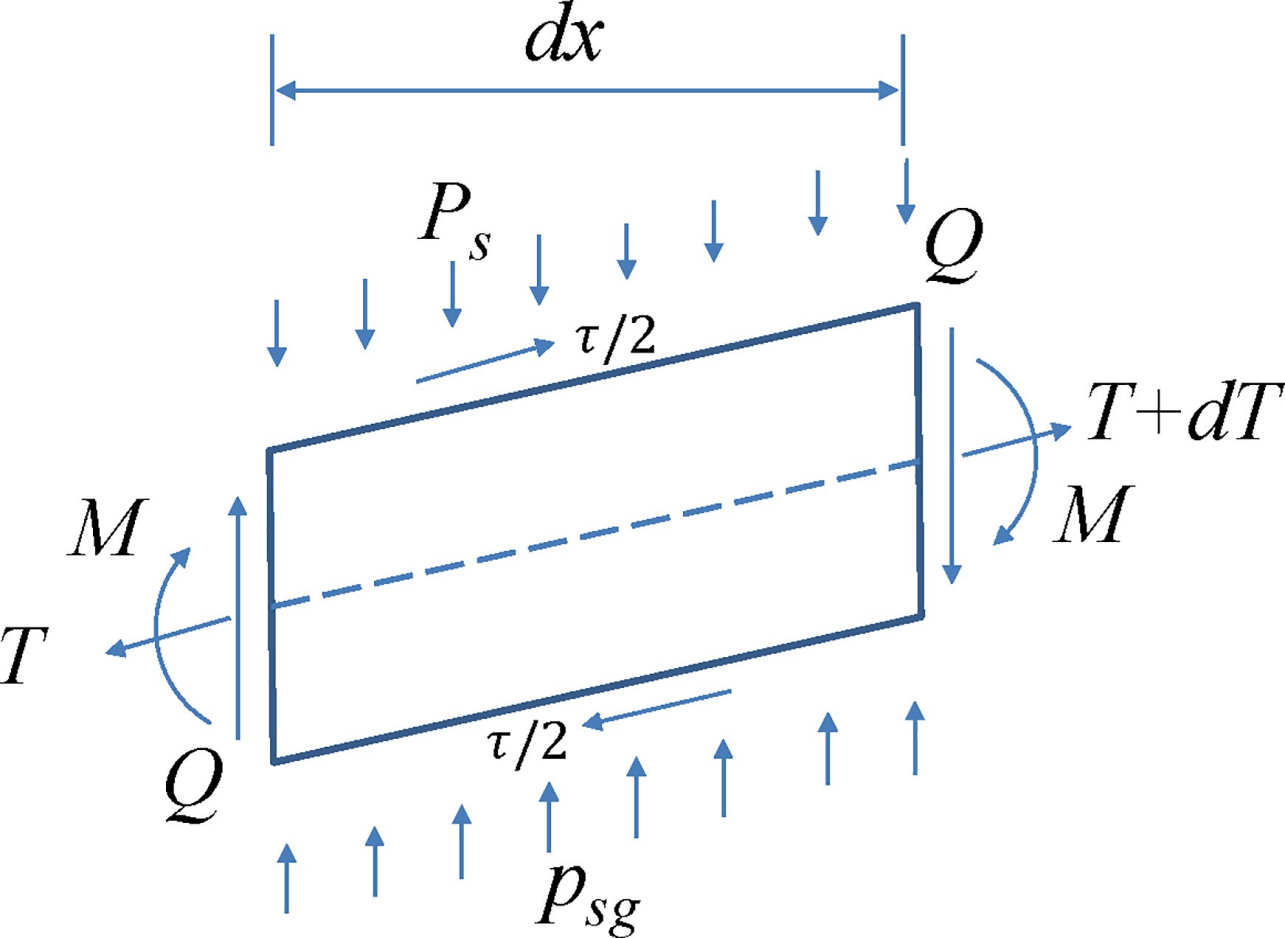
Analysis of the internal force of a soil element between piles for a GRPSE system with multi-layers of GR.

Figs 2 and 3 show the methodology used in modelling the stress of soil between the piles for a GRPSE system with multi-layers of GR using the Winkler elastic beam–foundation model. Considering a GR element (*dx*) (Fig 3), the equilibrium of force in vertical direction leads to,

$$Q-(Q+dQ)+p_{sg}bdx-P_{s}dx=0$$ (6A)

$$\frac{dQ}{dx}=bp_{sg}-P_{s}$$ (6B)

where *x* is the horizontal direction along GR, *b* is the calculated width of the beam on Winkler elastic foundation, and *Q* is the shear force on the GR. The differential equation for the deflection of the GR can be expressed as,

$$EI\frac{d^{2}\omega }{dx^{2}}=-M$$ (7)

where *EI* is the stiffness of the GR, *M* is the bending moment on the GR element, and *ω* is the vertical deflection of the GR. The moment equilibrium of GR element leads to,

$$M+dM-M-Qdx-T(-d\omega )-\frac{1}{2}P_{s}dx^{2}+\frac{1}{2}bp_{sg}dx^{2}=0$$ (8)

The tensile force of GR is caused by the friction between GR surface and soil, so *T*(*x*) = (*l*−*x*)*bτ*, Where *l* is the length of GR and *τ* is the frictional resistance per unit GR. Omitting the second-order differential yields [28],

dM−Qdx+Tdω=0 (9)

According to Winkler’s assumption that *p*_sg_(x,y) = k*s(x,y), Eq (10) can be obtained:

$$p_{sg}=ks=k\omega$$ (10)

Eq (11) can be obtained by simultaneous Eqs (6), (7), (9) and (10).

$$EI\frac{d^{4}\omega }{dx^{4}}-(l-x)b\tau \frac{d^{2}\omega }{dx^{2}}+b\tau \frac{d\omega }{dx}+bk\omega =P_{s}$$ (11)

where *k* is the coefficient of the subgrade soil between the piles of a GRPSE system, and *EI* is the flexural stiffness of the GR.

The boundary conditions of Eq (11) can be defined as: (1) At the outermost edges of the GR, the shear force and rotation angle are zero; (2) In the middle of the span, the bending moment and the deflection of the GR reach to their maximum values, i.e., *x* = 0,*Q* = 0,*ω*’ = 0, *x* =*r*_*p*_,*ω* = *ω*_max_; and (3) The bending moment and shear force of the GR at the edge of the pile cap are zero.

The maximum deflection of the GR, the maximum settlement of the soil between the piles (*S*_*s*_) and the corresponding subgrade stress (*p*_*sg*_) can be obtained by simultaneously solving the Eq (11) with boundary conditions. Thus, the maximum soil settlement between piles can be obtained,

$$S_{s\max}=\omega =\frac{P_{s}(r_{p}-r_{e})}{\sqrt[{4}]{16k^{3}EI}}A_{1}-\frac{P_{s}}{k}$$ (12)

where *r*_*p*_ is the radius of the pile cap, *r*_*e*_ is the equivalent treatment radius, *k* is the coefficient of the subgrade reaction, *EI* is the bending stiffness of the GR, *P*_*s*_ is the distributed load, and *A*_1_ is the subgrade coefficient of the embankment soil layer.

### 2.2. Optimization method for the bearing capacity of the soil between piles

#### 2.2.1. Optimization principle

**Fig 4:**
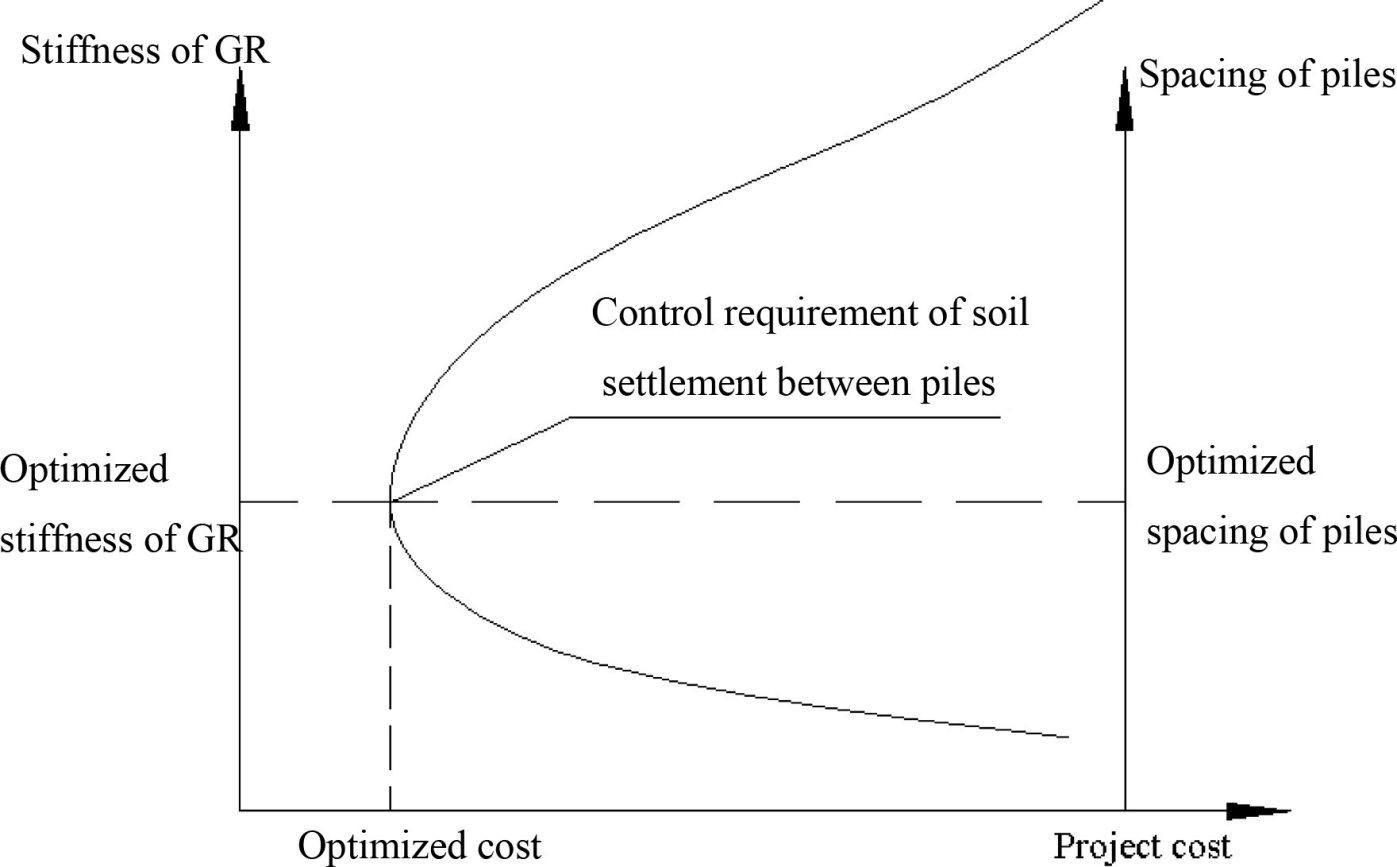
The schematic diagram showing the optimization principle in this study.

Based on the theoretical model developed in Section 2.1, the critical factors that govern the bearing capacity of the soil between piles are the spacing of the piles and the stiffness of the GR. However, reducing the pile spacing by using more piles or enhancing the performance of the GR could lead to the increase of project costs. Therefore, an optimization model was developed in this study to identify cost-effective pile layout and GR design which can potentially achieve the required settlement requirements of the soil between piles. The optimization principle was shown in Fig 4.

#### 2.2.2. Establish objective function

The optimized model was developed to identify the optimized stiffness of GR and spacing of piles which could lead to an optimized project cost. The parameters involved in the optimization process consist of pile length (*l*), pile spacing (*S*_*a*_), pile diameter (*d*), pile cap size (*a*), compression modulus (*E*_*c*_), pile–soil replacement rate (*m*), embankment cushion thickness (*h*_1_) and stiffness of GR (*EI*). The range of each parameter was selected based on design requirements and available literature [34]. The optimized project costs (*Q*_*p*_) can be obtained by min*zj*(*Q*_*p*_) through adjusting pile spacing and stiffness of GR. The constraints are given by,

$$\begin{array}{l} s_{\infty }\leq [s]\\ P_{p}\leq Q_{uk}/\gamma_{sp} \end{array}$$ (13)

where *s*_∞_ is the calculated settlement of the soil between piles, [*s*] is the maximum settlement required by the design, The calculation method is based on the research results of Chen et al. [35]., *P*_*p*_ is the embankment load, and *Q*_*uk*_ is the standard value of ultimate bearing capacity of pile. *γ*_*sp*_ is the partial coefficient of pile bearing capacity.

#### 2.2.3. Optimization procedure

The optimization procedure consists of the following steps:

1. Define initial pile layout (*S*_*a0*_) and stiffness of the GR (*EI*_*0*_). The tensile force of the GR shall meet the requirements of the technical code for composite foundation [36];
2. Calculate the bearing capacity (*Q*_*uk*_) and settlement of the soil between piles (*s*_∞_);
3. Adjust *S*_*a*_ and *EI* until *p*_*f*_ and *s*_∞_ meet the design requirements;
4. Estimate the costs of the GRPSE system (*Q*_*p*_) based on the values of *S*_*a*_ and *EI* obtained in Step (3);
5. Identify the optimized values of *S*_*a*_ and *EI* which lead to the optimized *Q*_*p*_ using model predictions optimization algorithm.

### 2.3. Field experimental and numerical analysis

#### 2.3.1. Project overview

In this section, the developed model was implemented to investigate the phase I of a highway project (South China coastal area). The field test research project has been approved by Zhuhai Tieke Geotechnical Engineering and Technology Corporation. This study focused on studying Section A2 of the highway. The test section is 160m long and was built on a soft foundation using a GRPSE system. The highway section number of the field experimental is K58 + 942 ~ k59 + 201, with a total length of 160 m. From the horizon down, the soil layers are filled soil layer, soft soil layer, silty clay layer and strongly weathered sandy layer. The filled soil layer thickness is 3.7–3.8 m. soft soil layer is 14–16 m thick and 17.7–19.8 m deep. The silty clay layer is 3–4.8 m thick and 20.7–24.6 m deep. The strongly weathered sand layer is about 20 m thick and 41-45m deep. According to the engineering investigation report prepared by the project (the report is prepared according to the “Code for investigation of geotechnical engineering” [37]), the distribution and mechanical properties of the soil layer are shown in Table 1.

**Table 1:** Soil distribution and mechanical properties.

Soil layer	Thickness (m)	soil depth(m)	Saturated bulk density (kN/m^3^)	Compression modulus (MPa)	Internal friction angle (°)	Cohesion (kPa)	Characteristic value of pile side resistance (kPa)
**Filled soil layer**	3.7–3.8	0–3.8	19	35	30	0	0
**Soft soil layer (silt)**	14.0–16.0	17.7–19.8	18 (8)	3	5	7	12
**Silty clay layer**	3.0	20.7–24.6	18 (8)	5	15	15	57
**Strongly weathered sandy soil layer**	20	41–45	18 (8)	80	45	3	150

**Fig 5:**
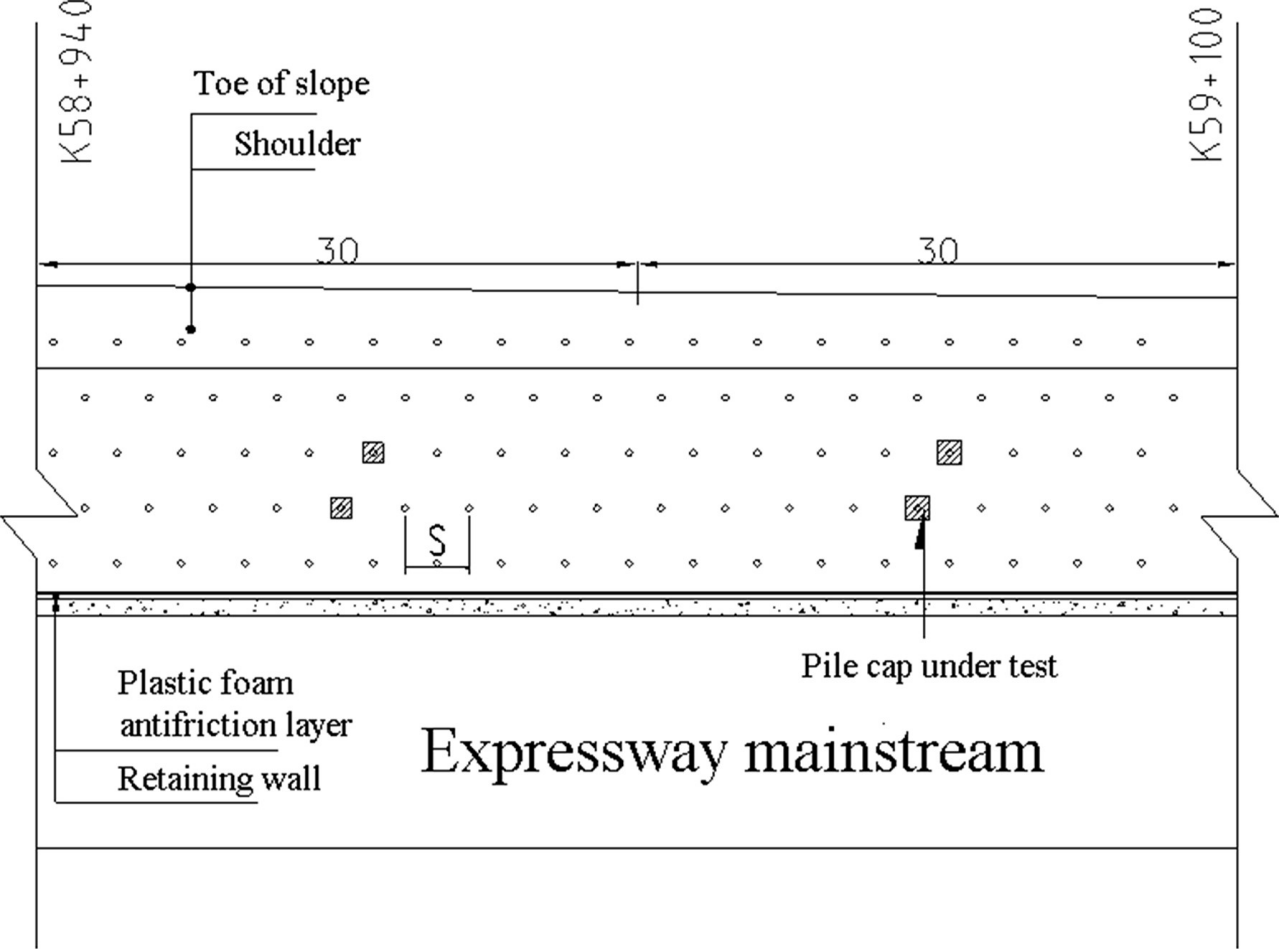
Embankment construction plan for Section A2 of the highway.

**Fig 6:**
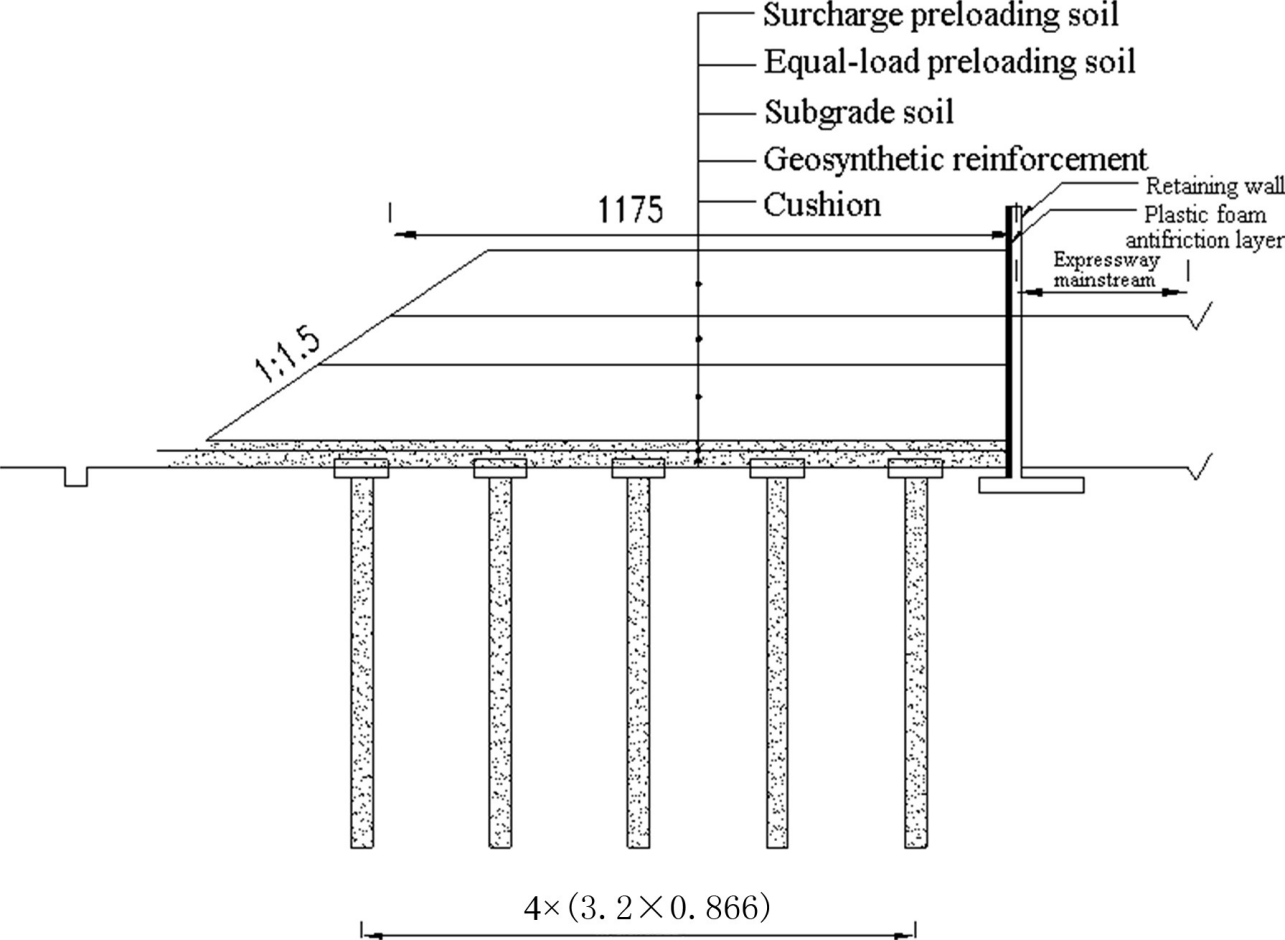
Embankment construction details for Section A2 of the highway.

**Fig 7:**
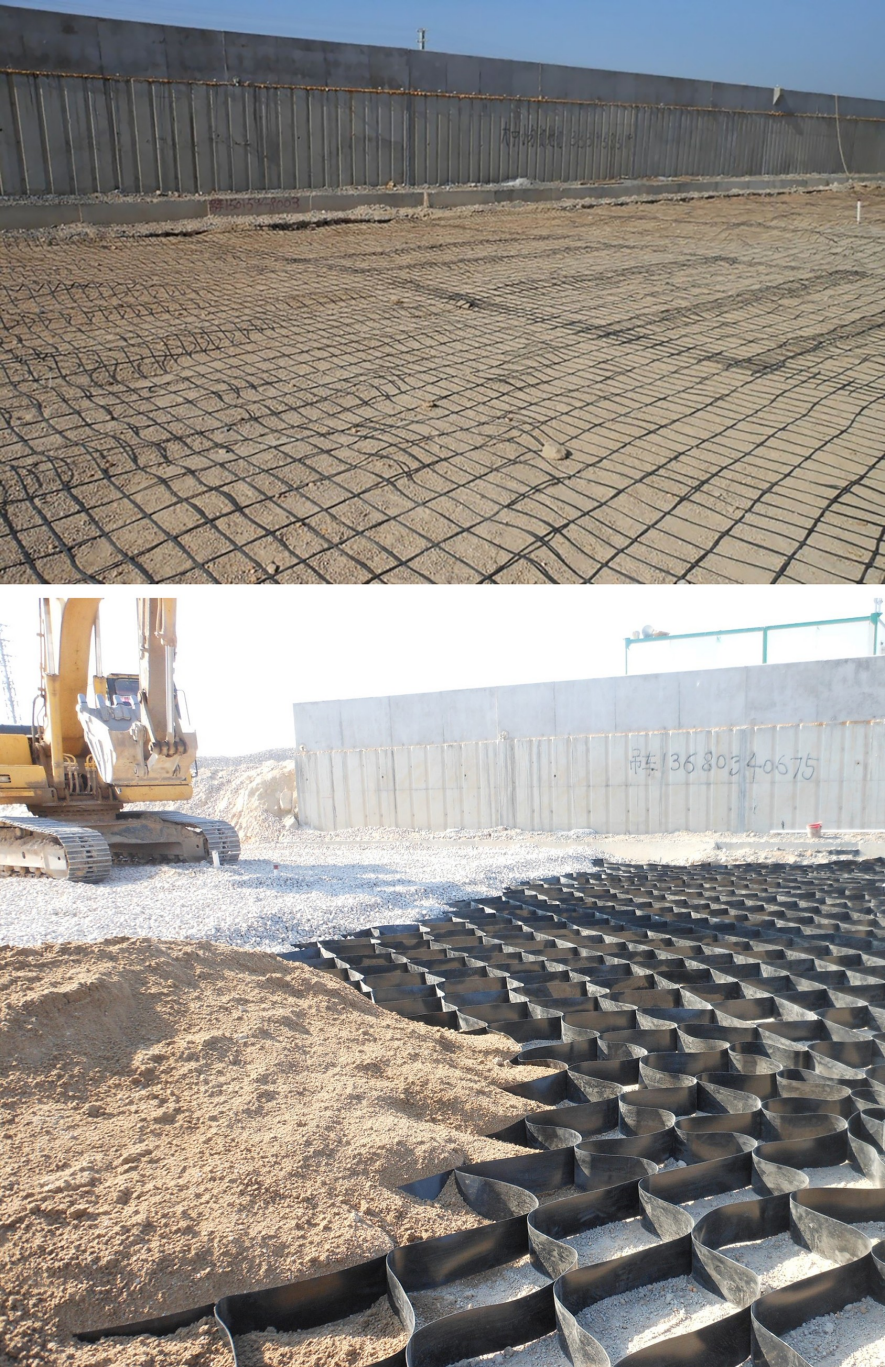
Site laying of the GR. (a)Geogrid laying. (b) Geocell laying.

Embankment construction plan for Section A2 of the highway is shown in Fig 5. The piles are arranged in a quincunx pattern. The diameter of the pipe pile is 0.3m with a spacing of 3m. The bearing stratum of the piles is the medium coarse sand with average pile length of around 20m and size of square pile cap ranging from 1.0 to 1.6m, Rigid connection is adopted between pile and pile cap. As shown in Fig 6, the filling height is 3.0–4.0 m and a geogrid and geocell reinforced cushion are used with the height of the geocell of 0.15m and the thickness of the cushion of 0.5 m. The schematic of the field laying is shown in Fig 7. The construction requirements of the geocell include levelling laying surface, ensuring main stress of the geocell in vertical direction, and the geocell in tension during laying and fixed with bamboo nails after laying.

#### 2.3.2. Field test setup

**Fig 8:**
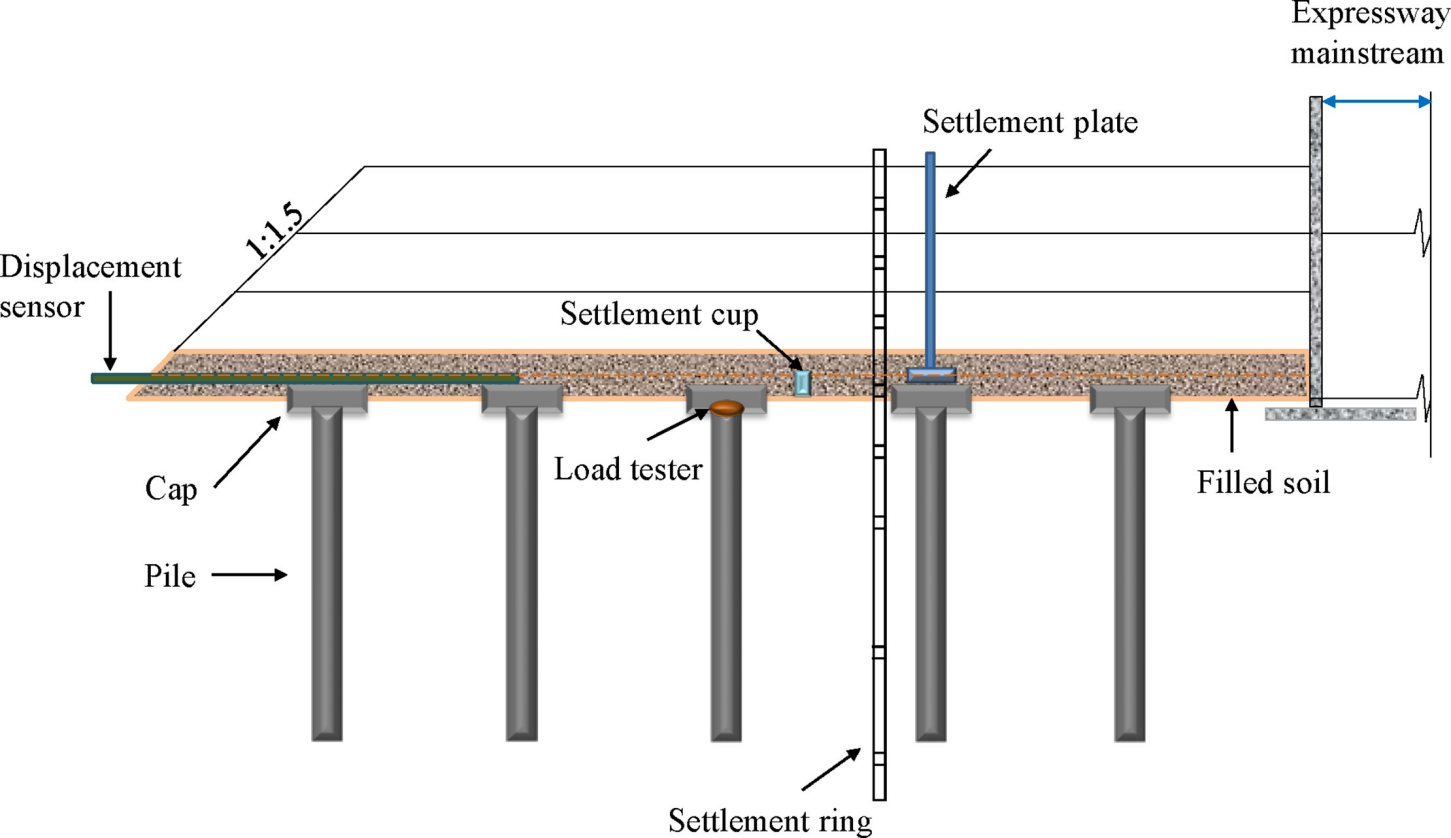
GRPSE system monitoring section and point layout diagram.

**Fig 9:**
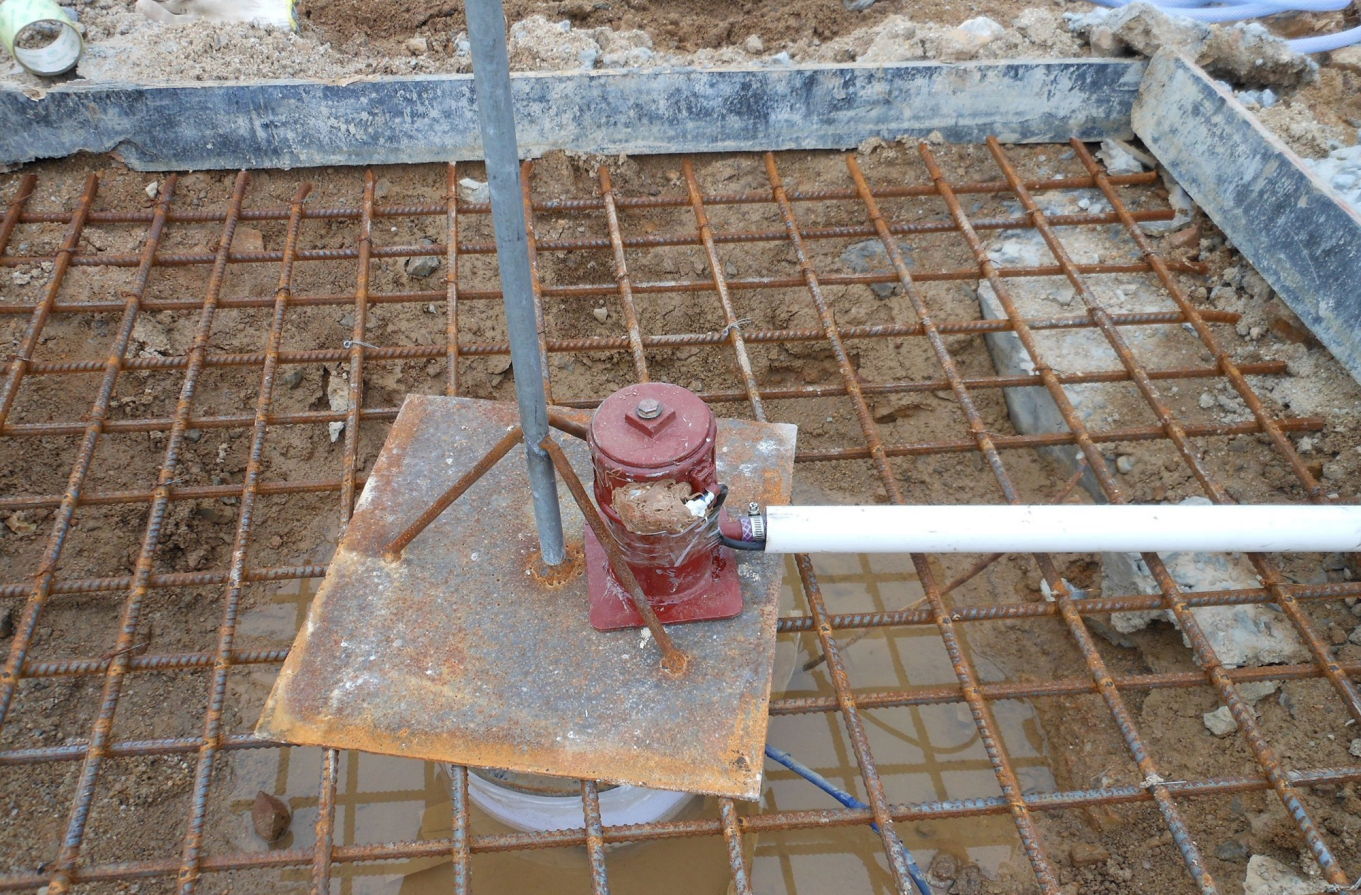
Installation of monitoring instruments.

As shown in Figs 8 and 9, a comprehensive monitoring system was developed to monitor the settlement and the bearing capacity of the GRPSE system. The theoretical results were validated by the measurement results. The monitoring system consists of the following components, and the details of monitoring points are shown in Table 2.

**Table 2:** Monitoring design parameters for each monitoring point.

No.	Location number of monitoring area	Length (m)	Filling height (m)	Pile and pile cap (m)			GR	
				Pile spacing	Pile cap	Clear spacing	Type	Layers
**1**	K58+940-K58+970	30	4.0	3.0	1.0	2.0	Geogrid	3
**2**	K58+970-K59+100	30	4.0	3.0	1.2	1.8	Geogrid	2
**3**	K59+000-K59+030	30	4.0	3.0	1.2	1.6	Geogrid	2
**4**	K59+030-K59+060	30	3.0	3.0	1.6	1.4	Geogrid	1
**5**	K59+060-K59+080	20	3.0	3.0	1.6	1.4	Geocell	1
**6**	K59+080-K59+100	20	3.0	3.0	1.6	1.4	Geocell	1

- Load cells: Install between the top of the pile and the cap to measure the load on the pile during the filling process;
- Soil settlement gauge: Install in the pile cap and buried into the soil to measure the settlement of the pile and the soil between the piles;
- Flexible strain gauge: Fixed on the grid of the GR to measure the tensile strain of the GR;
- Pore pressure gauge: buried into soil to measure the pore fluid pressure in the soil;
- Inclinometer tube: buried at the toe of the embankment to measure the horizontal displacement of the subgrade.

The installation process of the soil settlement gauge is as follows: put the water level gauge into the water tank, bury the water tank 1m outside the slope toe, and pour concrete to fix the water tank. Connect a water pipe between the two water tanks and pour clean water into the water tank so that the water surface is flush with the top of the tank.

The installation process of the earth pressure sensor is as follows: bury the earth pressure sensor in the designed position, wrap it with 30 cm fine sand and compact it. Lead the lead wire out of the slope toe, connect the instrument and set the instrument to zero.

#### 2.3.3. Numerical analysis

**Fig 10:**
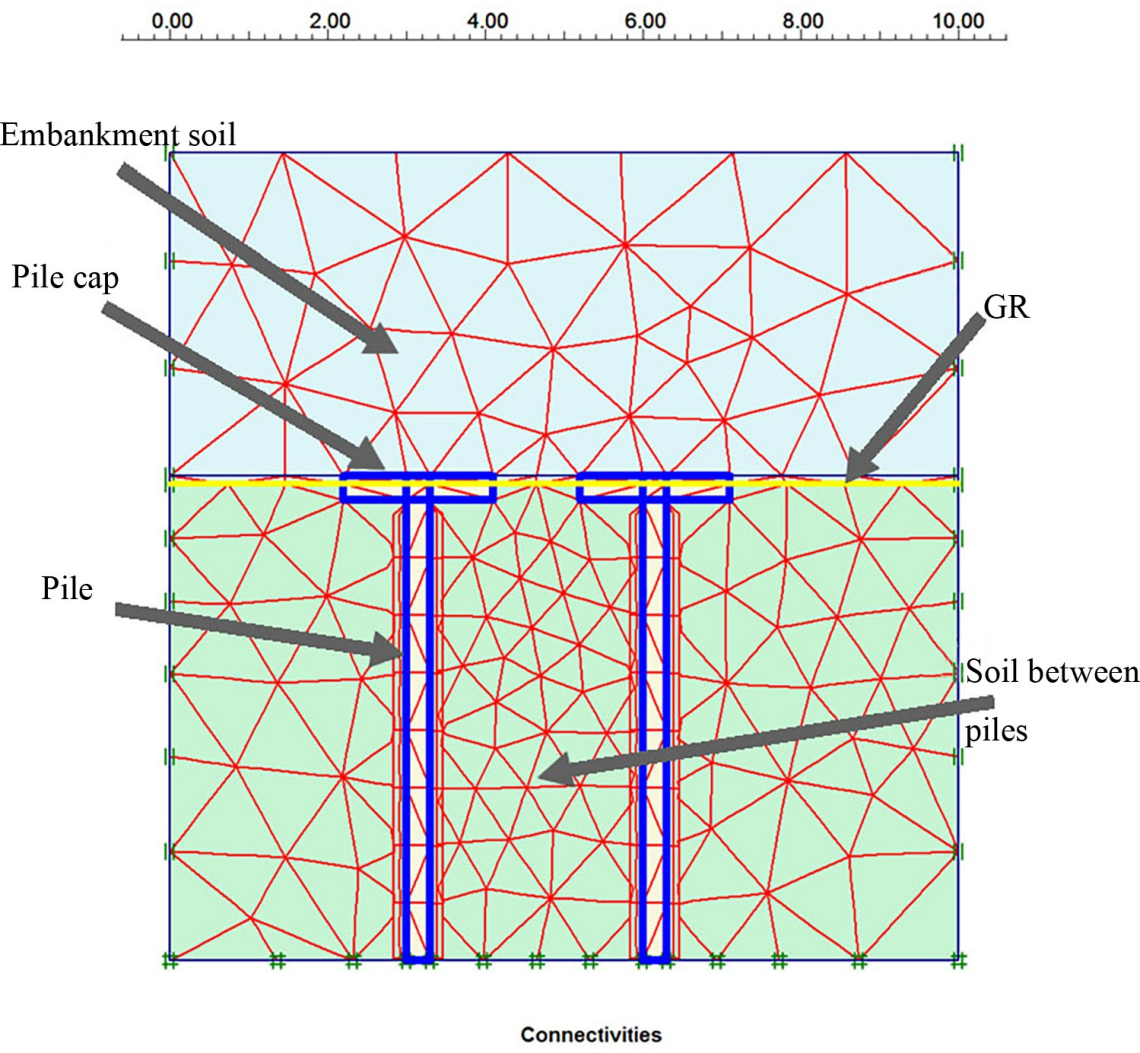
Finite element model.

The effect of pile spacing and geosynthetic reinforcement (GR) on the bearing capacity of the soil between piles was numerically analyzed. In the numerical model, the filling height is 3 m—4 m, the pile cap size is 1 m– 1.6 m, the pile length is 20 m, and the pile diameter is 0.3 m. The soil constitutive model adopts the modified Mohr-Coulomb model. GR adopts linear elastic model. Elastic model is adopted for pile cap and pile body. The values of the parameters used in numerical modelling are shown in Table 1 and Table 3. The left and right boundary displacements of GRPSE are set as vertical free and horizontal fixed. The upper boundary is displacement free and the lower boundary is displacement fixed. In the numerical model, a 15 node triangular element is used for finite element mesh generation. If the mesh thickness is set to "medium", a better result can be obtained, and the calculation time will be shortened. The model gridding is shown in Fig 10. The construction loading process is as follows:

- Calculate the self-weight stress of the soil layer;
- Set up pile element and GR element;
- Fill the embankment.

**Table 3:** Parameters used in numerical modelling.

Material	Cohesion (kPa)	Internal friction angle (°)	Deformation modulus (MPa)	Bulk density (kN/m^3^)	Unit	Constitutive model
**Embankment soil**	/	30	35	19	Triangle unit	Modified Mohr-Coulomb model
**GR**	15	/	2. 0 × 10^3^	15	Geogrid unit	Linear elastic model
**Pile / Pile cap**	/	/	1.5 × 10^4^	25	Linear elastic unit	Elastic model
**Soil between piles**	7	5	3	18	Triangle unit	Modified Mohr-Coulomb model

The numerical predications were validated using field testing data. In the process of numerical simulation, the tensile stiffness of geogrid and the bending stiffness of geocell are used for numerical analysis. The value of the tensile strength of the GR meets the requirements of the Technical code for composite foundation [36].

## 3. Results and discussion

### 3.1. Parametric studies

#### 3.1.1. Influence of pile spacing on the soil settlement between piles

After validation, a series parametric studies were carried out based on the settlement data of Section K58 + 970 –k59 + 000 located in the middle of the test section. This section mainly studies the influence of pile spacing and GR stiffness on soil settlement between piles. The bearing capacity of soil between piles is studied.

**Fig 11:**
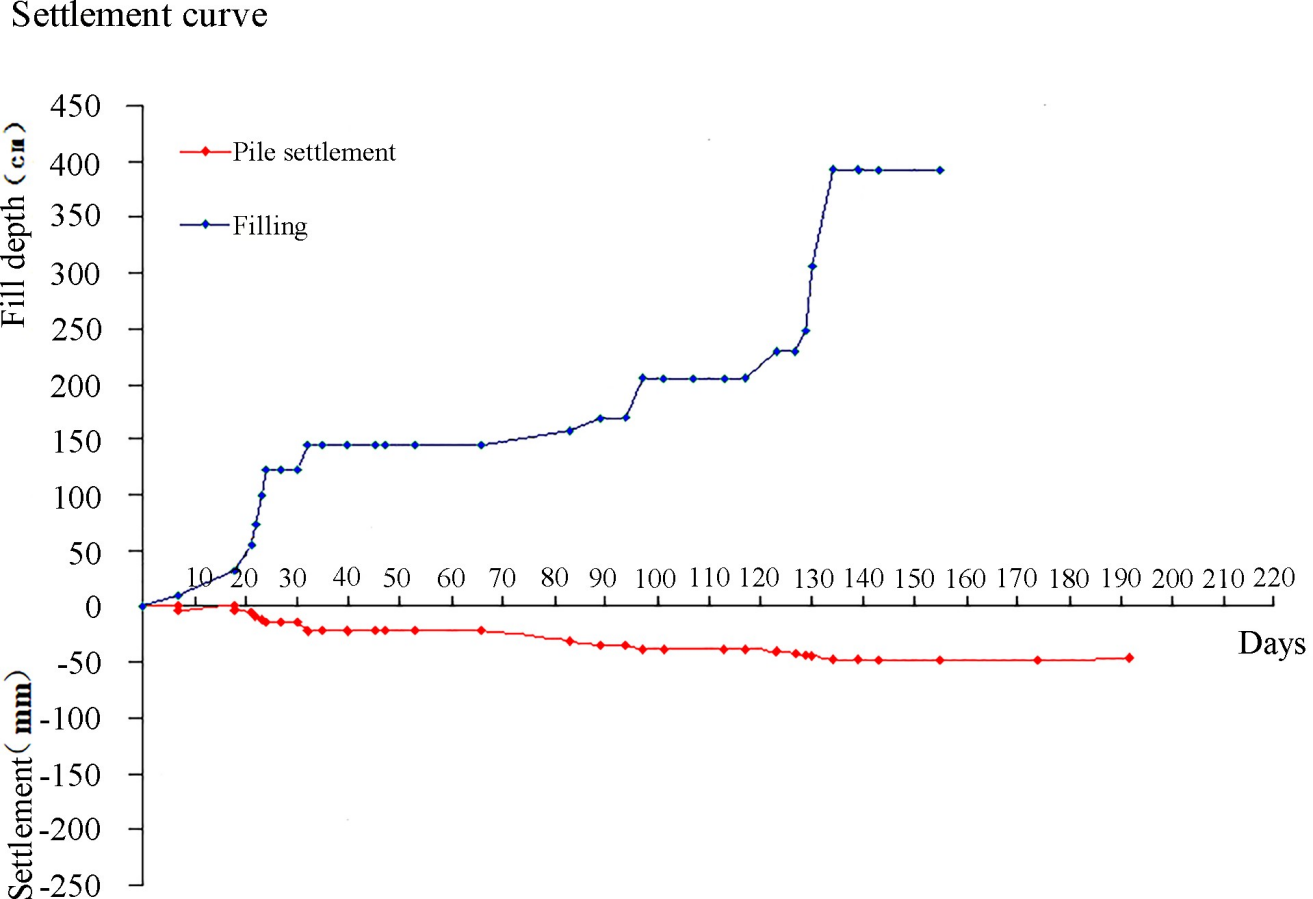
The time-dependent settlement of piles.

**Fig 12:**
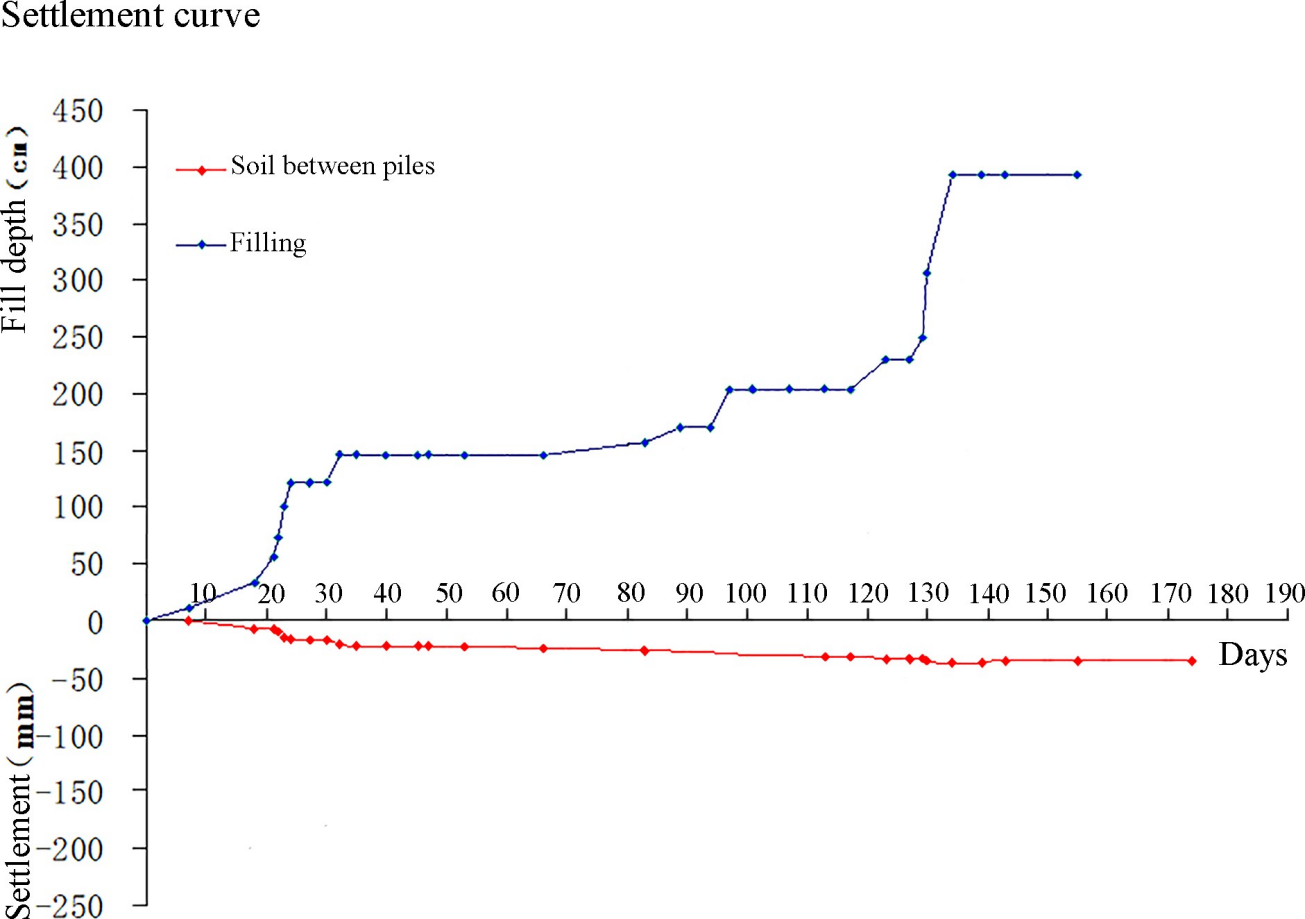
The time-dependent settlement of the soil between piles.

Figs 11 and 12 show the time-dependent settlement on the pile top and in the soil between piles, respectively. It shows that the settlement of the soil between the piles initially significantly increases with the increase of subgrade filling height, and then gradually decreases, and finally reaches to its steady-state. This indicates that the embankment settlement can be controlled by the construction process. In addition, it indicates that the maximum settlements on the piles and soil between piles are approximately 50mm and 60mm, respectively.

Table 4 shows settlements of pile top and soil between piles under different pile spacing (1.4, 1.6, 1.8, and 2.0 m). It can be seen that the decrease of pile spacing can significantly reduce the soil settlement between piles but has little influence in the settlement of pile cap. In addition, the pressure in soil between piles is very sensitive to the pile spacing, and the decrease of the pile spacing can significantly decrease the pressure in the soil between piles.

**Table 4:** Settlements of piles and soil between piles, and soil pressure under different pile spacing.

Pile spacing (m)	Settlement of soil between piles (mm)	Settlement of piles (mm)	Soil pressure (kPa)	Pile load (kN)
2.0	87	49	160	111.7
1.8	66	32	137	154.8
1.6	58	24	99	173.8
1.4	49	23	72	227.5

#### 3.1.2. Influence of the GR configuration on the soil settlement between piles

Table 5 shows the settlements of pile top and soil between piles, and soil pressure under different GR configurations. Two types of GR (*i*.*e*. geogrid and geocell) were investigated in this study. While the geogrid was used in Section 1–1–4–2 (i.e. K58 + 940 –K59 + 060), the geocell was implemented in Section 5–1–6–2 (K59 + 060 –K59 + 100). The results in Table 5 indicate that increasing the stiffness of GR using multiple layers of the GR can significantly decrease the settlement of piles and the soil between piles. For example, compared to 1-layer geogrid, the use of 2-layer geogrid could decrease the settlement of soil between piles, settlement of piles and soil pressure by 24%, 34% and 14%, respectively. In addition, the implementation of geocell could significantly enhance the performance of GRPSE. For example, the use of 1-layer geocell could reduce the settlement of soil between piles, settlement of piles and soil pressure by 37%, 55% and 70%, respectively, in comparison to that of 1-layer geogrid.

**Table 5:** Settlements of piles and soil between piles, and soil pressure under different GR configurations.

GR	Settlement of soil between piles (mm)	Settlement of piles (mm)	Soil pressure (kPa)	Pile load (kN)
**1-layer geogrid**	87	49	160	111.7
**2-layer geogrid**	66	32	137	154.8
**3-layer geogrid**	59	23	72	227.5
**1-layer geocell**	54	22	47	103.9

#### 3.1.3. Study on bearing capacity of soil between piles

**Fig 13:**
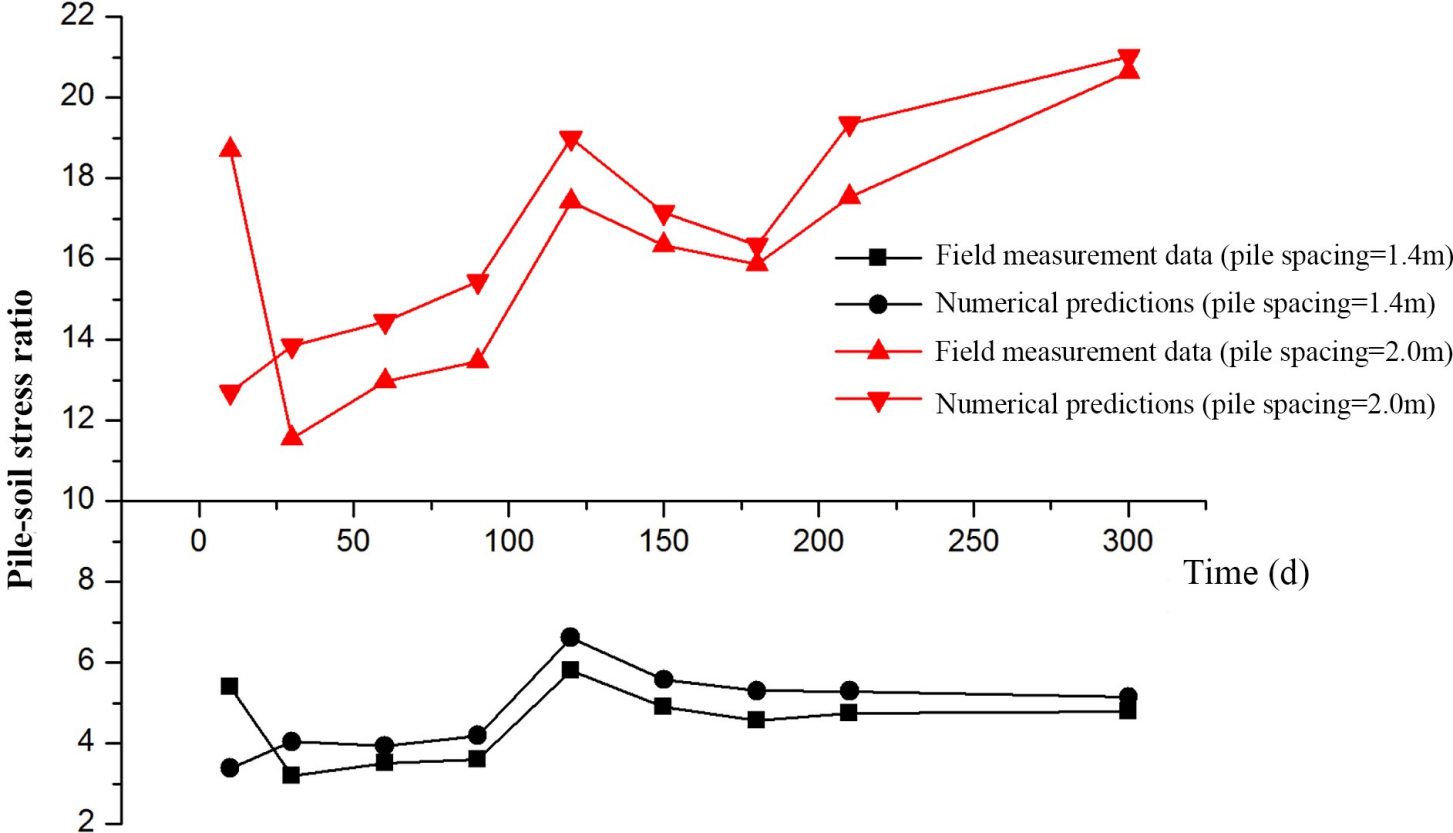
Effect of pile spacing on pile-soil stress ratio.

Fig 13 shows the influence of pile spacing on the pile-soil stress ratio. It demonstrates that the numerical predications agree with the field measurement data reasonably well. In addition, with the decrease of pile spacing, the load at the top of pile increases, and the pile-soil stress ratio also increases. The change of pile spacing will affect the pile-soil stress ratio. When the pile spacing increases from 1.4 m to 2.0 m, the pile-soil stress ratio increases. This figure shows that the soil load between piles is transferred to the pile by the membrane pulling action, and the load transfer action of GR is effective. Table 6 shows the effect of GR stiffness on pile-soil sharing ratio. The results show that the pile-soil sharing ratio increases with the increase of the GR stiffness, and geocell can provide a better load sharing outcome compared to GR. The numerical results show that the adjustment of pile spacing and GR stiffness can effectively improve the bearing capacity of soil between piles.

**Table 6:** Effect of GR stiffness on pile-soil sharing ratio.

GR stiffness (kN/m)	80 (single-layer geogrid)	160 (two-layer geogrid)	240 (three-layer geogrid)	120 (single-layer geocell)
**Numerical solution of load sharing ratio**	4.78	6.32	7.82	7.57
**Measured value of load sharing ratio**	5.41	6.38	7.64	7.29

#### 3.1.4. Study the stability of the GRPSE

Consolidation can cause the horizontal movement of soft soil. The stability of the embankment can be assessed by measuring the horizontal displacement of the bottom layer of the Section K58 + 950 using the inclinometer tubes. The measured time-dependent horizontal displacements are summarized in Table 7. The analysis of three-month horizontal displacement of substratum shows that the rate of horizontal displacement increment is around 0.12mm/day and the maximum horizontal displacement is around 24mm which is less than the allowable horizontal displacement (i.e. 40mm) [38].

**Table 7:** Maximum cumulative displacement of the substratum of the Section K58 + 950 (Depth = 13.5m).

Month	Jun	July	Aug	Sep
**Maximum horizontal displacement of substratum (mm)**	12.9	17.3	21.3	24.1

The above analysis indicates that the GRPSE has a significant effect on controlling the differential settlement of the embankment and adjusting the soil settlement between piles under the embankment. The pile spacing and stiffness of the GR are the most important factors affecting the soil settlement between piles. By adjusting the pile spacing and the stiffness of the GR, the bearing capacity of the soil between piles can be improved. It is confirmed that the calculation model for the soil settlement between piles and the optimization method for the bearing capacity of the soil between piles proposed in Section 2 are feasible.

### 3.2. Model validation

The purpose of this section is to validate the model predictions with measurement data obtained from field testing. According to the requirements of JGJ 94–2008 Technical code for building pile foundations [39], the bearing capacity of a pile can be obtained,

$$\begin{array}{l} R_{a}=\frac{1}{\gamma_{sp}}Q_{uk}\\ Q_{uk}=u\sum q_{sik}l_{i}+ap_{sk}A_{p} \end{array}$$ (14)

where *q*_*sik*_ is the ultimate flank resistance, *p*_*sk*_ is the ultimate end resistance, *Q*_*uk*_ is the vertical ultimate bearing capacity of a single pile, *K* is the safety factor (*K* = 2 in this study), *u* is the perimeter of the pile, *A*_*p*_ is the pile end area, *l*_*i*_ is the thickness of the *i-th* layer of the soil around the pile and *a* is the correction coefficient for the end resistance of a pile (*a* = 0.85 in this study). In order to avoid the instability failure of embankment, the embankment load borne by pile should be less than the bearing capacity of pile: *P*_*p*_≤*Q*_*uk*_/*γ*_*sp*_, Where *γ*_*sp*_ is the partial coefficient of pile bearing capacity.

Based on the field testing data as shown in Table 1, the value *p*_*sk*_ is determined to be 1503 kPa and the bearing capacity of a single pile can be obtained,

$$R_{a}=\frac{1}{\gamma_{sp}}Q_{uk}=\frac{(12\times 14+57\times 3+150\times 2)\times 0.3\times \pi +0.85\times 1\times 1503}{2}=940kN$$ (15)

Referring to the calculation method provided by Cao [40]., the pile top load is *P*_*p*_ *=* 826kN. The ultimate bearing capacity of pile foundation is: *R*_*a*_ = *Q*_*uk*_/*γ*_*sp*_ = 940*kN*>*P*_*p*_ = 826*kN*. The bearing capacity meets the requirements, and the embankment will not lose stability. Using pile spacing and stiffness of the GR as two optimization parameters, the model predicted settlements were compared with measurement data. The Eq 5 is used for predicting the maximum soil settlement between piles under single-layer of GR with consideration of the tensile stiffness of the GR only, while Eq 12 is for soil settlement prediction under multi-layer geogrid or geocell when the bending stiffness cannot be ignored. In the field experimental study, the settlement of GRPSE under different GR strength and pile spacing was investigated. The mechanical properties of GR used for settlement prediction are shown in Table 8. The stiffness of Geogrid and geocell is obtained by tensile test, which is the product specification. It shows that the predicated results agree with the measurement data reasonably well with an error ranging from 9%–13.5%. Based on the research results of Chen et al. [35], the maximum allowable settlement of the project is 20cm, so the calculation results meet the requirements of settlement. Most importantly, the developed model can produce a conservative outcome (*i*.*e*. predicted settlement > measured settlement), and therefore it is safe for its implementation in engineering practice. The difference in the settlement for two layers of geogrid with a pile spacing of 1.6m and one layer of geogrid with a pile spacing of 1.4m geogrid is relatively small (*i*.*e*. 3 mm). This indicates that the increase of GR stiffness can enhance the membrane effect. The bearing capacity of soil between piles is fully utilized. Therefore, the pile spacing can be increased.

**Table 8:** Comparing the predicted embankment settlements with the measured data.

Pile spacing (m)	GR	Stiffness (kN/m)	Predicted settlement (mm)	Measured settlement (mm)
1.8	2-layer geogrid	160	72	66
1.6	2-layer geogrid	160	64	58
1.4	1-layer geogrid	80	67	59
1.4	1-layer geocell	120	60	54

In addition, assuming the costs of a pipe pile and GR are ¥120/m and ¥10/m^2^, respectively, the total project costs under different combinations of pile spacing and GR stiffness were estimated as shown in Table 9. To fulfill the design requirements of the embankment and let the bearing capacity of a pile *R*_*a*_ = 940kN, an optimized project costs of ¥512,720 could be achieved by adopting a pile spacing of 1.8m and two layers of geogrid. Furthermore, in comparison to geogrid, the use of geocell could result in less project costs when pile spacing = 1.4m.

**Table 9:** Comparison of the pile quantities and costs for various embankment specifications.

Pile spacing (m)	GR	Bearing capacity of a pile (kN)	Total length of piles (m)	GR quantity (m^2^)	Total cost (¥)
1.8	2-layer geogrid	940	4034	2864	512,720
1.6	2-layer geogrid		4224	2258	529,460
1.4	1-layer geogrid		4556	2071	567,430
1.4	1-layer geocell		4260	1754	523,540

Therefore, based on the field tests, it is feasible to exploit the membrane effect by adjusting the pile spacing and the stiffness of the GR, which allows for full utilization of the bearing capacity of the soil between piles. The optimization method for the bearing capacity of the soil between piles introduced in this study can provide a more economical and reasonable arrangement scheme for the piles and GR.

## 4. Conclusions

This study presents a theoretical model to investigate the effects of pile spacing and stiffness of GR on the geotechnical performance of a GRPSE system. According to the theoretical model, the optimization method of bearing capacity of soil between piles is established. The model results and optimization method were validated using field testing data and numerical analysis. The following are major conclusions:

- The pile spacing and the stiffness of the GR are two critical factors that governing the bearing capacity of the soil between piles.
- The model results agree with the measurement data reasonably well with an error ranging from 9%– 13.5%. The developed model can generally produce a conservative outcome (i.e. predicted settlement > measured settlement), and therefore it is safe for its implementation in engineering practice.
- optimization method differs from the traditional design method, which is mainly based on load control. By controlling the settlement of soil between piles, the membrane effect is exerted and the utilization ratio of bearing capacity of soil between piles is improved. This method can effectively save the project cost.
- In comparison to geogrid, the use of geocell could result in less project costs.
- The settlement of the soil between the piles initially significantly increases with the increase of subgrade filling height, and then gradually decreases before reaching to its steady-state. This indicates that the embankment settlement can be controlled by the construction process.
- Although the decrease of pile spacing can significantly reduce the soil settlement between piles, the decrease has little influence in the settlement of pile cap.
- The pressure in soil between piles is very sensitive to the pile spacing, and the decrease of the pile spacing can significantly decrease the soil stress between piles.
- Increasing the stiffness of GR using multiple layers of the GR can significantly decrease the settlement of piles and the soil between piles. For example, compared to 1-layer geogrid, the use of 2-layer geogrid could decrease the settlement of soil between piles, settlement of piles and soil pore pressure by 24%, 34% and 14%, respectively.
- The implementation of geocell could significantly enhance the performance of GRPSE. For example, the use of 1-layer geocell could reduce the settlement of soil between piles, settlement of piles and soil pressure by 37%, 55% and 70%, respectively, in comparison to that of 1-layer geogrid.
- By comparing the engineering quantity and cost, the optimal pile arrangement scheme is found. The scheme meets the requirements of bearing capacity and settlement of embankment. The scheme can provide valuable reference for solving the problems related to engineering cost and engineering design.

## Supporting information

S1 Notation (DOCX)
