# Supplementary material for: Calculation model and bearing capacity optimization method for the soil settlement between piles in geosynthetic-reinforced pile-supported embankments based on the membrane effect
Source: PLoS One. 2021 Aug 16;16(8):e0256190. doi: 10.1371/journal.pone.0256190 (PMC8366969; doi:10.1371/journal.pone.0256190)
Supplement: S1 Notation — (DOCX) [file pone.0256190.s001.docx]

# Notation

The following symbols are used in this paper:

$r_{p}$= radius of the pile cap(m);

$r_{e}$= equivalent treatment radius(m);

$S_{s\max}$= maximum settlement of the soil between piles(m);

$\varepsilon_{g}$= tensile strain at the GR(dimensionless);

$E_{g}$= elastic modulus of the GR(Pa);

$S_{a}$= pile spacing(m);

$p_{sg}$= maximum reaction force between piles(Pa);

$P_{pg}$= upper load of pile cap(Pa)

$k$= coefficient of the subgrade reaction(dimensionless);

$T$= tension of the GR(N);

$P_{s}$= uniform load of the upper soil(Pa);

$Q$= section shear force of the GR(N);

$b$= calculated width of beam on Winkler elastic foundation (m);

$EI$= flexural stiffness of the GR(dimensionless);

$\omega$= vertical deflection of the GR(m);

$s$= foundation deformation(m);

$A_{1}$= subgrade coefficient of the embankment soil layer. (dimensionless);

$l$= pile length(m);

$d$= pile diameter(m);

$a$= pile cap size(m);

$E_{c}$= compression modulus of the GRPSE(Pa);

$m$= pile-soil replacement rate(dimensionless);

$h_{1}$= embankment cushion thickness(m);

$s_{\infty}$= calculated settlement(m);

$\left[s\right]$= maximum settlement allowed by the design(m);

*P_p_* = embankment load(N);

$Q_{uk}$= standard value of ultimate bearing capacity of pile(N);

$\gamma_{sp}$= partial coefficient of pile bearing capacity(dimensionless);

$R_{a}$= bearing capacity of a single prestressed pipe pile(Pa);

$q_{sik}$= [standard value of the ultimate flank resistance](http://dict.cnki.net/dict_result.aspx?searchword=%e6%9e%81%e9%99%90%e4%be%a7%e9%98%bb%e5%8a%9b%e6%a0%87%e5%87%86%e5%80%bc&tjType=sentence&style=&t=standard+value+of+ultimate+flank+resistance)(Pa) ;

$p_{sk}$ = standard value of the ultimate end resistance(Pa);

$u$= perimeter of the pile(m);

$A_{p}$= pile end area(m^2^);

$l_{i}$= thickness of layer $i$ of the soil around the pile(m);

$a$ = correction coefficient for the pile end resistance(dimensionless);

$x$= horizontal direction along GR(m).
